# Measuring parameters of aerobic function during exercise: One protocol to rule them all?

**DOI:** 10.1113/EP092732

**Published:** 2025-03-22

**Authors:** David C. Poole, Harry B. Rossiter

**Affiliations:** ^1^ Departments of Kinesiology and Anatomy & Physiology Kansas State University Manhattan Kansas USA; ^2^ Institute of Respiratory Medicine and Exercise Physiology The Lundquist Institute for Biomedical Innovation at Harbor‐UCLA Medical Center Torrance California USA

**Keywords:** cardiopulmonary exercise testing, gas exchange threshold, kinetics parameter estimation, maximum oxygen uptake, oxygen uptake kinetics

1

Rigorous determination of key parameters of oxidative function, namely the maximum O_2_ uptake (${\dot{V}}_{O_{2}\max}$), ${\dot{V}}_{O_{2}}$ kinetics and work efficiency, provides invaluable information regarding the O_2_ transport system and integrated cerebral–pulmonary–cardiovascular–muscular function in health and disease. For instance, these parameters relate mechanistically to lifespan, health span, physical exercise performance and exercise (in)tolerance. Moreover, in patient populations, these parameters can stratify individuals for heart transplant procedures, assess surgical outcomes and present sensitive measures of treatment toxicity (e.g., doxorubicin and anthracyclines in cancer) as well as discriminate therapeutic benefits of imposed countermeasures to disease or age‐related debilitation. The ramp‐incremental exercise test, with gas exchange measurements, was originally devised to provide information on each of these parameters in a single laboratory visit (Whipp et al., 1981). Indeed, this approach forms the basis of clinical cardiopulmonary exercise testing guidelines worldwide (e.g., Palange et al., 2018). However, over time, it became apparent that ${\dot{V}}_{O_{2}}$ kinetics and work efficiency varied considerably with exercise intensity (e.g., Özyener et al., 2001; Rossiter, 2011), and therefore new approaches were needed to evaluate these diverse aspects of oxidative function in a single exercise test.

To that end, Santana et al. (2025) are to be applauded for their recognition of the importance of streamlining laboratory testing to facilitate the measurement of multiple oxidative parameters (i.e., ${\dot{V}}_{O_{2}\mathrm{peak}}$, ${\dot{V}}_{O_{2}}$ kinetics and, although not discussed therein, work efficiency). However, prior to the implementation of the precise testing protocol advocated by Santana et al. (2025), multiple checks and balances should be considered based upon sound logic and scientific rigour.

Specifically:

1. There needs to be consideration of the on–off asymmetry that normally attends moderate‐ versus severe‐intensity exercise (e.g., Özyener et al., 2001). This unavoidably leads to the very slow overall time constants observed in recovery from ${\dot{V}}_{O_{2}\mathrm{peak}}$, where there is clearly ${\dot{V}}_{O_{2}}$ slow component behaviour (e.g., extant in Figure 4b of Santana et al. (2025)). This inaccuracy can be ameliorated by the application of a two‐component model for the recovery analyses (Özyener et al., 2001).
2. Use of 30% predicted ${\dot{V}}_{O_{2}}$ reserve to determine moderate‐intensity ${\dot{V}}_{O_{2}}$ kinetics is infeasible for patient populations with moderate to severe impairments in O_2_ transport and/or utilization. For example, patients with heart failure or chronic obstructive pulmonary disease can have ${\dot{V}}_{O_{2}\mathrm{peak}}$ values that are less than 30% predicted ${\dot{V}}_{O_{2}}$ reserve. Also, in severe heart failure, even unloaded pedalling can be attended by a raised arterial lactate and is therefore no longer in the moderate‐intensity domain, complicating kinetics analysis (Özyener et al., 2001). While the participants in Santana et al.’s study fell within the range of ‘normal’ aerobic fitness (based on group mean values, males achieved 100% and females achieved 106% of predicted ${\dot{V}}_{O_{2}\mathrm{peak}}$), their kinetics were measured at 36–37% of predicted ${\dot{V}}_{O_{2}\mathrm{peak}}$. These ${\dot{V}}_{O_{2}}$ values could easily exceed the gas exchange threshold (GET) in a deconditioned population (a guideline value to identify deconditioning is GET < 40%). Therefore, consideration of the time constant for moderate‐intensity ${\dot{V}}_{O_{2}}$ kinetics using this protocol also requires confirmation that the participant is exercising below GET.
3. Repeated transitions are requisite for accurate kinetics parameter analysis from breath‐by‐breath data (e.g., Benson et al., 2017). Such procedures would likely have removed the poor fit and systematic bias evident in Figure 4.
4. The ${\dot{V}}_{O_{2}\mathrm{peak}}$ protocol should ensure that ${\dot{V}}_{O_{2}\max}$ is validated and therefore measured accurately (Poole & Jones, 2017).
5. Data need to be carefully screened for aberrant behaviour. Specifically, that ${\dot{V}}_{O_{2}}$ remains at peak for 40 s into ‘recovery’ in Figure 2 stands in opposition to a legion of prior observations and mechanistic underpinnings of exercise energetics (e.g., Özyener et al., 2001).
6. Finally, Santana et al. report that ${\dot{V}}_{O_{2}\mathrm{peak}}$ is much higher in males (per kg) than females, and yet their ${\dot{V}}_{O_{2}}$ on‐kinetics is far slower. This does not cohere with the compelling weight of published evidence to the contrary (see Poole & Jones, 2012).

Attention to these and additional considerations will lessen the ‘streamlined’ nature of the testing protocol proposed by Santana et al. (2025) but is essential for ensuring accurate parameter estimation and to enhance translation of their approach to patients and other defined populations.

A fundamental tenet of science is that it be self‐correcting with expeditious rectification limiting pernicious sequelae. Today we are privileged to exist in a space and time where, as in this instance, science can be corrected in real time. We hope that such an exercise, as demonstrated by this Viewpoint, will be of interest and pertinent to scientists at all stages of their careers and help reinforce confidence that science is indeed self‐correcting.

## AUTHOR CONTRIBUTIONS

Both authors have read and approved the final version of this manuscript and agree to be accountable for all aspects of the work in ensuring that questions related to the accuracy or integrity of any part of the work are appropriately investigated and resolved. All persons designated as authors qualify for authorship, and all those who qualify for authorship are listed.

## CONFLICT OF INTEREST

Harry Rossiter is a visiting Professor at the University of Leeds, UK.

## FUNDING INFORMATION

No funding was received for this work.
